# Supplementary material for: The role of N6-methyladenosine (m6A) RNA methylation modification in kidney diseases: from mechanism to therapeutic potential
Source: PeerJ. 2025 Aug 27;13:e19940. doi: 10.7717/peerj.19940 (PMC12398287; doi:10.7717/peerj.19940)
Supplement: Supplemental Information 2 [file peerj-13-19940-s002.docx]

**Identification of studies via databases and registers**

Records removed *before screening*:

Duplicate records removed (n = 179)

Records marked as ineligible by automation tools (n = 49)

Records removed for other reasons (n = 0)

Records identified from*:

Databases (n = 457)

Registers (n = 0)

**Identification**

Records screened

(n = 253)

Records excluded**

(n = 150)

Reports sought for retrieval

(n = 150)

Reports not retrieved

(n = 10)

**Screening**

Reports assessed for eligibility

(n = 140)

Reports excluded:

Study design mismatch (n = 5)

Incomplete data (n = 5)

Duplicate data (n = 3)

Studies included in review

(n = 127)

Reports of included studies

(n = 127)

**Included**

*Consider, if feasible to do so, reporting the number of records identified from each database or register searched (rather than the total number across all databases/registers).

**If automation tools were used, indicate how many records were excluded by a human and how many were excluded by automation tools.

Source: Page MJ, et al. BMJ 2021;372:n71. doi: 10.1136/bmj.n71.

This work is licensed under CC BY 4.0. To view a copy of this license, visit <https://creativecommons.org/licenses/by/4.0/>
